# Memory-Enhancing Activity of Palmatine in Mice Using Elevated Plus Maze and Morris Water Maze

**DOI:** 10.1155/2012/357368

**Published:** 2012-11-01

**Authors:** Dinesh Dhingra, Varun Kumar

**Affiliations:** Department of Pharmaceutical Sciences, Guru Jambheshwar University of Science and Technology, Haryana, Hisar 125001, India

## Abstract

The present study was designed to evaluate the effect of palmatine on memory of Swiss young male albino mice. Palmatine (0.1, 0.5, 1 mg/kg, *i.p.*) and physostigmine (0.1 mg/kg, *i.p.*) per se were administered for 10 successive days to separate groups of mice. Effect of drugs on learning and memory of mice was evaluated using elevated plus maze and Morris water maze. Brain acetylcholinesterase activity was also estimated. Effect of palmatine on scopolamine- and diazepam-induced amnesia was also investigated. Palmatine (0.5 and 1 mg/kg) and physostigmine significantly improved learning and memory of mice, as indicated by decrease in transfer latency using elevated plus maze, and decrease in escape latency during training and increase in time spent in target quadrant during retrieval using Morris water maze. The drugs did not show any significant effect on locomotor activity of the mice. Memory-enhancing activity of palmatine (1 mg/kg) was comparable to physostigmine. Palmatine (1 mg/kg) significantly reversed scopolamine- and diazepam-induced amnesia in mice. Palmatine and physostigmine also significantly reduced brain acetylcholinesterase activity of mice. Thus, palmatine showed memory-enhancing activity in mice probably by inhibiting brain acetylcholinesterase activity, through involvement of GABA-benzodiazepine pathway, and due to its antioxidant activity.

## 1. Introduction

Dementia, the commonest form (accounting for approximately 60% of all cases) of which is Alzheimer's disease (AD), mainly affects older people and it is estimated that, by 2050, more than 115 million people will have dementia [1]. AD is a neurodegenerative disorder characterized by cognitive and memory deterioration, progressive impairment of activities of daily living, and a multiplicity of behavioural and psychological disturbances [2]. The primary causes of AD appear to be (i) decreased cholinergic activity; (ii) deposition of amyloid-beta peptides in the brain; (iii) oxidative stress. Acetylcholinesterase (AChE) plays a key role in the regulation of the cholinergic system and hence, inhibition of AChE has emerged as one of the most promising strategies for the treatment of AD. One of the major therapeutic strategies is to inhibit the AChE, and hence, to increase the acetylcholine level in the brain [3]. The imbalance between the generation of free radicals and antioxidants has also been claimed to be a cause of AD [4]. 

Palmatine is a quaternary protoberberine alkaloid. It is typically yellow in color and is an active constituent of a number of plants, such as *Coptidis rhizoma *[5], and so forth. Palmatine has been reported to possess sedative [6] and antioxidant activities [5]. It has been also shown to be inhibitor of beta-site amyloid precursor protein-cleaving enzyme 1 (BACE 1), acetyl- and butyrylcholinesterases [5]. Thus, palmatine has potential for the management of dementia. So the present study was designed to investigate the effect of palmatine on the learning and memory of mice by employing behavioral models.

## 2. Materials and Methods

### 2.1. Experimental Animals

Swiss male albino mice, weighing around 20–25 g, were purchased from Disease Free Small Animal House, Lala Lajpat Rai University of Veterinary and Animal Sciences, Hisar (Haryana). Since estrogens (female sex hormones) have been found to have effect on memory, we excluded female mice and used only male mice for the study [7]. Animals were housed separately in groups of 8 per cage (Polycarbonate cage size: 29 × 22 × 14 cm) under laboratory conditions with alternating light and dark cycle of 12 h each. The animals had free access to food and water. The animals were kept fasted 2 h before and 2 h after drug administration. The animals were acclimatized for at least five days before behavioural experiments which were carried out between 09:00 and 17:00 h. The experimental protocol was approved by Institutional Animals Ethics Committee (IAEC) and animal care was taken as per the guidelines of Committee for the Purpose of Control and Supervision of Experiments on Animals (CPCSEA), Ministry of Environment and Forests, Government of India (Registration no. 0436). 

### 2.2. Drugs and Chemicals

Palmatine and scopolamine hydrobromide (Sigma-Aldrich, St. Louis, USA); physostigmine, acetylcholine iodide, acetylthiocholine iodide, and 5,5′-dithiobis-2-nitrobenzoic acid (Hi-Media Laboratories, Mumbai); diazepam (Calmpose injection, Ranbaxy Laboratories Ltd., Gurgaon, India).

### 2.3. Selection of Doses

Doses of various drugs were selected on the basis of literature, that is, 0.4 mg/kg for scopolamine, 1 mg/kg for diazepam [8], 0.1 mg/kg for physostigmine [9], 0.1 and 1 mg/kg for palmatine [8].

### 2.4. Vehicle

Palmatine was suspended in 10% Tween 80 in normal saline. Scopolamine hydrobromide was dissolved in normal saline. Injection of diazepam was diluted in normal saline.

### 2.5. Models Employed for Evaluation of Memory Enhancing Activity in Mice

#### 2.5.1. Elevated Plus Maze

The procedure, technique, and end point for testing learning and memory were followed as per the parameters described earlier [8, 10, 11]. The elevated plus maze for mice consisted of two open arms (16 cm × 5 cm) and two covered arms (16 cm × 5 cm × 15 cm) extended from a central platform (5 cm × 5 cm) and the maze was elevated to a height of 25 cm from the floor. On the first day, each mouse was placed at the end of an open arm, facing away from the central platform. Transfer latency (TL) was defined as the time taken by the animal to move from the open arm into one of the covered arms with all its four legs. TL was recorded on the first day (i.e., 10th day of drug administration) for each animal. If the animal did not enter into one of the covered arm within 90 sec, it was gently pushed into one of the two covered arms and TL was assigned as 90 sec. The mouse was allowed to explore the maze for another 2 minutes and then returned to its home cage. Retention of this learned-task (memory) was examined 24 h (11th day) after the first day trial. 

#### 2.5.2. Morris Water Maze

The procedure, technique, and end point for testing memory were followed as per the parameters described earlier [12, 13]. Briefly, Morris water maze-(MWM) for mice consisted of a circular pool (60 cm in diameter, 25 cm in height) filled to a depth of 20 cm with water maintained at 25°C. The water was made opaque with nontoxic white colored dye. The tank was divided into four equal quadrants with the help of two threads, fixed at right angle to each other on the rim of the pool. A submerged platform (with top surface 6 cm × 6 cm and painted in white) was placed inside the target quadrants (Q4 in present study) of this pool 1 cm below surface of water. The position of platform was kept unaltered throughout the training session. Each animal was subjected to four consecutive trials each day with a gap of 5 min for four consecutive days (starting from 6th day of drug administration to 9th day), during which they were allowed to escape on to the hidden platform and to remain there for 20 s. During the training session, the mouse was gently placed in the water between quadrants, facing the wall of pool with drop location changing for each trial, and allowed 120 sec to locate submerged platform. If the mouse failed to find the platform within 120 s, it was guided gently on to the platform and allowed to remain there for 20 s. Escape latency (EL) is the time taken by the animal to move from the starting quadrant to find the hidden plateform in the target quadrant. EL was recorded on the 6th day to 9th day for each animal. Each animal was subjected to training trials for four consecutive days, the starting position was changed with each exposure as mentioned below and target quadrant (Q4 in the present study) remained constant throughout the training period.

 Day1 Q1 Q2 Q3 Q4.
 Day2 Q2 Q3 Q4 Q1.
 Day3 Q3 Q4 Q1 Q2.
 Day4 Q4 Q1 Q2 Q3.

On the fifth day (i.e., 10th day of drug administration), the platform was removed and mouse was placed in any of the three quadrants and allowed to explore the target quadrant for 300 s. Mean time spent in all the three quadrants that is, Q1, Q2, and Q3 was recorded. The mean time spent in the target quadrant in search of the missing platform was noted as index of retrieval or memory. The observer always stood at the same position. Care was taken not to disturb the relative location of water maze with respect to other objects in the laboratory.

#### 2.5.3. Measurement of Locomotor Activity

To rule out the effects of the drugs on motor activity, horizontal locomotor activities of control and test animals were recorded for a period of 5 min using Medicraft Photoactometer, Model number 600-4D (INCO, Ambala, India). The photoactometer consisted of a square arena (30 × 30 × 25 cm) with wire mesh bottom, in which the animal moves. Six lights and six photocells placed in the outer periphery of the bottom in such a way that a single mouse can block only one beam. Technically its principle is that a photocell is activated when the rays of light falling on the photocells are cut off by animals crossing the beam of light. As the photocell is activated, a count is recorded. The photocells are connected to an electronic automatic counting device which counts the number of “cut offs.” 

### 2.6. Biochemical Estimation

#### 2.6.1. Collection of Brain Sample

Immediately after behavioural testing (retrieval) on elevated plus maze, animals were sacrificed by cervical dislocation under light anaesthesia with diethylether. The whole brain was carefully removed from the skull. For preparation of brain homogenate, the fresh whole brain was weighed and transferred to a glass homogenizer and homogenized in an ice bath after adding 10 volumes of phosphate buffer (pH 8, 0.1 M). The homogenate was centrifuged using refrigerated centrifuge at 3000 rpm for 10 min at 4°C and the resultant cloudy supernatant liquid was used for the estimation of brain acetylcholinesterase activity.

#### 2.6.2. Brain Acetylcholinesterase Activity

Brain acetylcholinesterase was estimated using the method of Ellman et al. [14]. Briefly, 0.4 mL of brain homogenate was added to a test tube containing 2.6 mL of phosphate buffer. 0.1 mL DTNB reagent was added to the above mixture and absorbance was noted at 412 nm. 0.02 mL of acetylcholine iodide solution was added and again absorbance was noted 15 min thereafter. Change in absorbance per min was calculated.

The rate of hydrolysis of substrate was calculated using following formula:

 
*R*  = change in absorbance/min⁡×5.74 × 10^−4^/*C*0,
 
*R*  = rate of hydrolysis of acetylcholine iodide/min/mg tissue,
 
*C*0  = weight of tissue homogenate in mg/mL.

### 2.7. Experimental Design

#### 2.7.1. Groups for Elevated Plus Maze

**Group 1 to 5**

Normal saline, palmatine (0.1, 0.5 and 1 mg/kg, *i.p.*), and physostigmine (0.1 mg/kg, *i.p.*), respectively, were administered for 11 successive days. TL was recorded 30 minutes after the drug administration on 10th day (learning) and retention was examined on 11th day.

**Group 6 and 7**

Normal saline and palmatine (1 mg/kg, *i.p.*), respectively, were injected for 11 successive days. On 10th day, TL was recorded 45 min after the injection. On the 11th day, scopolamine was injected (0.4 mg/kg, *i.p.*) 30 min after injection of palmatine and TL was recorded 45 min after the injection of scopolamine.

**Group 8 and 9**

Normal saline and palmatine (1 mg/kg, *i.p.*), respectively, were injected for 11 successive days. On 10th day, TL was recorded 45 min after the injection. On the 11th day, diazepam was injected (0.4 mg/kg, *i.p.*) 30 min after injection of palmatine and TL was recorded 45 min after the injection of diazepam.

#### 2.7.2. Groups for Morris Water Maze

**Groups 10 to 14**

Normal saline, palmatine (0.1, 0.5 and 1 mg/kg, *i.p.*), and physostigmine (0.1 mg/kg, *i.p.*), respectively, were administered for 10 successive days. Escape latency (EL) was recorded 45 min after drug administration from 6th day to 9th day. On 10th day, time spent in target quadrant (TSTQ) was noted 45 min after the drug administration.

**Group 15 and 16**

Normal saline and palmatine (1 mg/kg, *p.o.*), respectively, were injected for 10 successive days. EL was recorded 45 min after drug administration from 6th day to 9th day. On 10th day, scopolamine was injected 30 min after injection of palmatine and TSTQ was noted 45 min after the injection of scopolamine.

**Group 17 and 18**

Normal saline, palmatine (1 mg/kg, *p.o.*), respectively, were injected for 10 successive days. EL was 45 min after drug administration from 6th day to 9th day. On 10th day, diazepam (1 mg/kg, *i.p.*) was injected 30 min after injection of palmatine and TSTQ was noted 45 min after the injection of diazepam.

#### 2.7.3. Measurement of Locomotor Activity

Locomotor activity was measured 24 h after performing water maze test in mice of groups 10 to 14 using photoactometer (INCO, Ambala).

### 2.8. Statistical Analysis

All the results are expressed as Mean ± S.E.M. Data were analyzed by analysis of variance (ANOVA) followed by Tukey's post hoc test in Graph Pad Instat package, version 3.05. *P* < 0.05 was considered as significant.

## 3. Results

### 3.1. Effect of Palmatine and Other Drugs Employed on Transfer Latency (TL) of Mice

Palmatine and physostigmine administered for 10 successive days did not significantly affect TL of mice on 10th day (learning) as compared to the control group. But palmatine (0.5 and 1 mg/kg, *i.p.*) and physostigmine (0.1 mg/kg, *i.p.*) significantly decreased TL in mice on 11th day (memory) as compared to the control group, thus showed significant memory enhancing activity. The lowest dose of palmatine (0.1 mg/kg, *i.p.*) did not significantly decrease TL of mice on 11th day as compared to vehicle treated control group. Scopolamine (0.4 mg/kg, *i.p.*) and diazepam (1 mg/kg, *i.p.*) significantly increased TL in mice, indicating its amnesic effect. Palmatine (1 mg/kg, *i.p.*) significantly reversed scopolamine-induced and diazepam-induced memory impairment in mice as compared to respective scopolamine and diazepam treated groups (Table 1). 

### 3.2. Effect of Palmatine and Other Drugs Employed on Escape Latency (EL) and Time Spent in Target Quadrant (TSTQ) of Mice Using Morris Water Maze

Palmatine (0.5 and 1 mg/kg, *i.p.*) and physostigmine (0.1 mg/kg, *i.p.*) significantly decreased EL of mice on 9th day and increased TSTQ by mice on 10th day as compared to the control group, thus showed significant improvement of learning and memory. The lowest dose of palmatine (0.1 mg/kg, *i.p.*) did not significantly decrease EL or increase TSTQ as compared to vehicle treated control group. Scopolamine (0.4 mg/kg, *i.p.*) and diazepam (1 mg/kg, *i.p.*) significantly increased EL and decreased TSTQ by mice, indicating their amnesic effects. Palmatine (1 mg/kg, *i.p.*) significantly reversed scopolamine-induced and diazepam-induced learning and memory impairment in mice as compared to respective scopolamine and diazepam treated groups (Tables 2 and 3). 

### 3.3. Effect of Palmatine and Physostigmine on Brain Acetyl Cholinesterase (AChE) Activity in Mice

**Figure 1:**
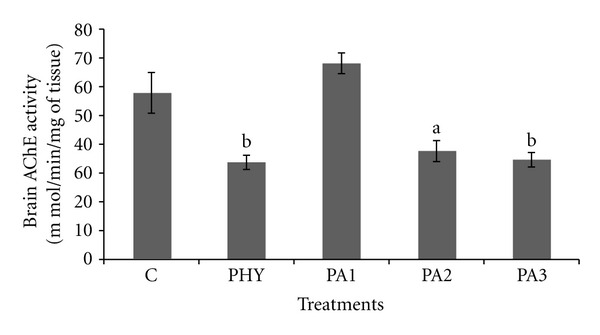
Effect of palmatine and physostigmine on brain AChE activity of mice. *n* = 8 in each group.   Values are expressed as Mean ± SEM. Data was analyzed by one-way ANOVA followed by Tukey's Post-hoc test. *F*(4, 33) = 14.736; *P* < 0.0001; ^a^ *P* < 0.05 as compared to control; ^b^ *P* < 0.01 as compared to control; C = Control; PHY = Physostigmine (0.1 mg/kg); PA1 = Palmatine (0.1 mg/kg); PA2 = Palmatine (0.5 mg/kg); PA3 = Palmatine (1 mg/kg).

Administration of palmatine (0.5 mg/kg and 1 mg/kg) and physostigmine for 11 consecutive days produced a significant decrease in brain AChE activity as compared to control group. The lowest dose of palmatine 0.1 mg/kg did not produce significantly decrease in AChE activity as compared to control group (Figure 1).

### 3.4. Effect of Palmatine and Physostigmine on Locomotor Activity of Mice

Palmatine and physostigmine used in the present study did not significantly affect the spontaneous locomotor activities of mice as compared to the respective control groups (Table 4).

## 4. Discussion

In the present study, palmatine (0.5 and 1 mg/kg, *i.p.*) administered for 10 successive days showed significant memory enhancing effect in mice. This is the first study showing memory enhancing activity of palmatine in mice. Elevated plus maze and Morris water maze were employed as behavioral models for evaluation of learning and memory. These models are widely employed for evaluating the effect of drugs on learning and memory [10, 12]. In elevated plus maze, decrease in transfer latency on 2nd day (i.e., 24 h after the first trial) indicated improvement of memory and viceversa. In Morris water maze, a decrease in escape latency during training and increase in time spent in target quadrant during retrieval indicated improvement of learning and memory respectively; and *vice versa*. Palmatine did not show any significant change in locomotor functions of mice as compared to the vehicle treated control, so this did not produce any motor effects. Thus, memory enhancing effect of palmatine is specific and not false positive. Out of the two effective doses of palmatine (0.5 and 1 mg/kg, *i.p.*), higher dose (1 mg/kg) produced better memory enhancing effect in mice (*P* < 0.01) as compared to the lower dose (*P* < 0.05) in both the behavioural models employed, hence the higher dose (1 mg/kg) was employed for elucidating the probable mechanisms of memory enhancing activity.

Central cholinergic system plays a major role in regulation of cognitive function [15]. Drugs that reduce cholinergic function such as muscarinic receptor antagonist scopolamine produce amnesia in laboratory animals. In the present study, scopolamine and diazepam significantly impaired memory of mice. Memory impairment effect of diazepam has been reported in the literature [11]. Palmatine (1 mg/kg, *i.p.*) administered for 10 successive days to separate groups of mice significantly reversed scopolamine-induced amnesia and diazepam-induced amnesia in mice. Benzodiazepines produce amnesia in laboratory animals by activation of benzodiazepine receptors and GABAergic system [16, 17]. Flumazenil (benzodiazepine-receptor antagonist) and beta-carbolines (benzodiazepine inverse agonist) have been demonstrated to reverse benzodiazepine-induced amnesia [18]. Reversal of scopolamine- and diazepam-induced amnesia by palmatine indicated the possible facilitation of cholinergic transmission or GABA-benzodiazepine pathway. Palmatine (1 mg/kg) also significantly reduced brain AChE activity in mice as compared to the control group. This suggests that the memory enhancing effect of palmatine might be due to inhibition of AChE, leading to increase in brain levels of acetylcholine. This is supported by an earlier study where palmatine showed inhibition of acetylcholinesterase activity [5]. Acetylcholine is considered to be one of the important neurotransmitter involved in the regulation of cognitive functions. Cognitive dysfunction has been shown to be associated with impaired cholinergic transmission and the facilitation of central cholinergic transmission resulting in improved memory. Moreover, selective loss of cholinergic neurons in certain brain parts appeared to be a characteristic feature of senile dementia [19]. The degeneration and dysfunction of cortical cholinergic neurons is closely associated with cognitive deficits of AD [20]. Thus, the drugs which enhance cholinergic function can be used for treatment of dementia closely related to AD. Physostigmine (0.1 mg/kg, *i.p.*) injected for 10 successive days significantly improved memory of mice. Memory enhancement activity of physostigmine has been well reported in the literature. Physostigmine, a cholinesterase inhibitor, could improve memory in normal subjects [21] as well as in patients with dementia [22].

The memory enhancing activity of palmatine is also supported by its beta-site amyloid precursor protein-cleaving enzyme 1 (BACE 1) inhibiting property [5]. BACE1 is the major beta-secretase to cleave the beta-amyloid precursor protein to generate beta-amyloid. Oxidative stress has been shown to affect amyloid-beta generation in the AD pathogenesis. Upregulation of BACE 1 gene transcription by oxidative stress may contribute to the pathogenesis of AD [23]. Palmatine has also been reported to possess antioxidant activity [5]. Thus, palmatine produced significant memory enhancing effect in mice probably due to its antioxidant property by virtue of which susceptible brain cells get exposed to less oxidative stress resulting in reduced brain damage and improvement of neuronal function. 

In conclusion, palmatine showed memory enhancing activity in mice probably by inhibiting brain acetylcholinesterase activity, through involvement of GABA-benzodiazepine pathway and due to its antioxidant activity. 

## Figures and Tables

**Table 1:** Effect of palmatine and other drugs employed on transfer latency (TL) of mice using elevated plus maze.

Treatments	Dose (kg)^−1^	TL (sec) on 10th day	TL (sec) on 11th day
Control (vehicle) for 10 days	10 mL	20.25 ± 2.27	17.13 ± 1.24
Physostigmine for 10 days	0.1 mg	16.38 ± 1.39	8.25 ± 0.88^b^
Scopolamine	0.4 mg	19.25 ± 2.16	28.25 ± 1.85^b^
Diazepam	1 mg	19.14 ± 2.11	24.57 ± 2.30^a^
Palmatine for 10 days	0.1 mg	18.37 ± 1.73	14.13 ± 1.73
Palmatine for 10 days	0.5 mg	15.87 ± 1.72	9.88 ± 0.83^a^
Palmatine for 10 days	1 mg	15.75 ± 2.30	8.75 ± 0.75^b^
Palmatine for 10 days + scopolamine on 10th day	1 mg + 0.4 mg	15.14 ± 1.98	15.57 ± 1.2^c^
Palmatine for 10 days + diazepam on 10th day	1 mg + 1 mg	20.75 ± 2.20	17 ± 1.50^d^

*n* = 8 in each group. Values are expressed as Mean ± SEM. Data was analyzed by one-way ANOVA followed by Tukey's post-hoc test.

*F*(8, 60) = 1.417; *P* = 0.02079 (10th day);

*F*(8, 60) = 21.034; *P* < 0.0001 (11th day);

^a^ *P* < 0.05 as compared to control;

^b^ *P* < 0.01 as compared to control;

^c^ *P* < 0.001 as compared to scopolamine treated group;

^d^ *P* < 0.01 as compared to diazepam treated group.

**Table 2:** Effect of palmatine and other drugs employed on escape latency (EL) of mice using Morris water maze.

Treatments	Dose (kg)^−1^	EL (sec) Day-6	EL (sec) Day-7	EL (sec) Day-8	EL (sec) Day-9
Control (vehicle) for 10 days	10 mL	105.90 ± 3.28	96.34 ± 3.44	78.38 ± 3.70	54.19 ± 3.44
Physostigmine for 10 days	0.1 mg	104.31 ± 3.06	101.25 ± 2.51	74.09 ± 2.45	33.5 ±2.66^c^
Scopolamine	0.4 mg	106.62 ± 18.16	87.66 ± 3.75	69.63 ± 3.49	44.44 ± 2.81^b^
Diazepam	1 mg	105.96 ± 2.59	91.66 ± 2.48	69.47 ± 3.21	42.56 ± 2.52^a^
Palmatine for 10 days	0.1 mg	103.93 ± 2.95	100.93 ± 3.13	78.09 ± 4.01^b^	44.22 ± 2.98
Palmatine for 10 days	0.5 mg	100 ± 3.25	88.38 ± 3.84	61.56 ± 2.98^c^	32.09 ± 2.4^b^
Palmatine for 10 days	1 mg	99.75 ± 2.80	75.43 ± 3.54^b^	49.78 ± 2.31	26.06 ± 1.54^c^
Palmatine for 10 days + scopolamine on 10th day	1 mg + 0.4 mg	107.29 ± 3.01	83.69 ± 3.48	54.75 ± 2.26	28.13 ± 1.33^d^
Palmatine for 10 days + diazepam on 10th day	1 mg + 1 mg	109.90 ± 2.23	85.21 ± 2.84	55.81 ± 3.39	29.91 ± 1.59^e^

*n* = 8. Values are expressed as Mean ± SEM. Data was analyzed by one-way ANOVA followed by Tukey's post-hoc test.

*F*(8, 279) = 1.264; *P* = 0.2625 (day 6);

*F*(8, 279) = 7.147; *P* < 0.0001 (day 7);

*F*(8, 279) = 11.079; *P* < 0.0001 (day 8);

*F*(8, 279) = 14.251; *P* < 0.0001 (day 9);

^a^ *P* < 0.05 as compared to control;

^b^ *P* < 0.01 as compared to control;

^c^ *P* < 0.001 as compared to control;

^d^ *P* < 0.001 as compared to scopolamine treated group;

^e^ *P* < 0.01 as compared to diazepam treated group.

**Table 3:** Effect of palmatine and other drugs employed on time spent in target quadrant of mice using Morris water maze.

Treatments	Dose (kg)^−1^	Time spent (sec) in target quadrant (10th day)
Control (vehicle) for 10 days	10 mL	94.38 ± 4.50
Physostigmine for 10 days	0.1 mg	125.88 ± 6.98^a^
Scopolamine	0.4 mg	65.50 ± 4.04^a^
Diazepam	1 mg	68.13 ± 5.16^a^
Palmatine for 10 days	0.1 mg	85.25 ± 4.07
Palmatine for 10 days	0.5 mg	119.63 ± 5.33^a^
Palmatine for 10 days	1 mg	124.12 ± 5.26^b^
Palmatine for 10 days + scopolamine on 10th day	1 mg + 0.4 mg	109 ± 7.78^c^
Palmatine for 10 days + diazepam on 10th day	1 mg + 1 mg	96.5 ± 5.29^d^

*n* = 8. Values are expressed as Mean ± SEM. Data was analyzed by one-way ANOVA followed by Tukey's post-hoc test.

*F*(8, 63) = 17.197; *P* < 0.0001;

^a^ *P* < 0.05 as compared to control;

^b^ *P* < 0.01 as compared to control;

^c^ *P* < 0.001 as compared to scopolamine treated group;

^d^ *P* < 0.05 as compared to diazepam treated group.

**Table 4:** Effect of palmatine and physostigmine on locomotor activity of mice.

Treatment for 10 days	Dose (kg)^−1^	Locomotor activity counts/5 min
Control	10 mL	297.43 ± 9.5
Physostigmine	0.1 mg	310.14 ± 10.64
Palmatine	0.1 mg	281.38 ± 12.41
Palmatine	0.5 mg	294 ± 10.69
Palmatine	1 mg	302.75 ± 5.12

*n* = 8 in each group. Values are expressed as Mean ± SEM. Data was analyzed by one-way ANOVA followed by Tukey's post-hoc test.

*F*(4, 33) = 1.152; *P* = 0.3497.
